# Immunogenicity and Protection Efficacy of a Naked Self-Replicating mRNA-Based Zika Virus Vaccine

**DOI:** 10.3390/vaccines7030096

**Published:** 2019-08-23

**Authors:** Zifu Zhong, João Paulo Portela Catani, Séan Mc Cafferty, Liesbeth Couck, Wim Van Den Broeck, Nina Gorlé, Roosmarijn E. Vandenbroucke, Bert Devriendt, Sebastian Ulbert, Lieselotte Cnops, Johan Michels, Kevin K. Ariën, Niek N. Sanders

**Affiliations:** 1 Laboratory of Gene Therapy, Department of Nutrition, Genetics and Ethology, Faculty of Veterinary Medicine, Ghent University, Heidestraat 19, 9820 Merelbeke, Belgium; 2 Cancer Research Institute (CRIG), Ghent University, 9000 Ghent, Belgium; 3 Department of Morphology, Faculty of Veterinary Medicine, Ghent University, Salisburylaan 133, 9820 Merelbeke, Belgium; 4 Barriers in Inflammation Lab, Department of Biomedical molecular biology, Faculty of Sciences, Ghent University, Technologiepark-Zwijnaarde 71, 9052 Zwijnaarde, Belgium; 5 VIB Center for Inflammation Research, VIB, Technologiepark-Zwijnaarde 71, 9052 Zwijnaarde, Belgium; 6 Laboratory of Immunology, Department of Virology, Parasitology and Immunology, Faculty of Veterinary Medicine, Ghent University, Salisburylaan 133, 9820 Merelbeke, Belgium; 7 Department of Immunology, Fraunhofer Institute for Cell Therapy and Immunology, Perlickstr. 1, 04103 Leipzig, Germany; 8 Unit of Neglected Tropical Diseases, Department of Clinical Sciences, Institute of Tropical Medicine, 2000 Antwerp, Belgium; 9 Virology Unit, Institute of Tropical Medicine, 2000 Antwerp, Belgium; 10 Department of Biomedical Sciences, University of Antwerp, 2000 Antwerp, Belgium

**Keywords:** self-replicating mRNA, Zika virus vaccine, type I interferon response, IFNAR1 knockout mice

## Abstract

To combat emerging infectious diseases like Zika virus (ZIKV), synthetic messenger RNAs (mRNAs) encoding viral antigens are very attractive as they allow a rapid, generic, and flexible production of vaccines. In this work, we engineered a self-replicating mRNA (sr-mRNA) vaccine encoding the pre-membrane and envelope (prM-E) glycoproteins of ZIKV. Intradermal electroporation of as few as 1 µg of this mRNA-based ZIKV vaccine induced potent humoral and cellular immune responses in BALB/c and especially IFNAR1^-/-^ C57BL/6 mice, resulting in a complete protection of the latter mice against ZIKV infection. In wild-type C57BL/6 mice, the vaccine resulted in very low seroconversion rates and antibody titers. The potency of the vaccine was inversely related to the dose of mRNA used in wild-type BALB/c or C57BL/6 mice, as robust type I interferon (IFN) response was determined in a reporter mice model (IFN-β^+/Δβ-luc^). We further investigated the inability of the sr-prM-E-mRNA ZIKV vaccine to raise antibodies in wild-type C57BL/6 mice and found indications that type I IFNs elicited by this naked sr-mRNA vaccine might directly impede the induction of a robust humoral response. Therefore, we assume that the efficacy of sr-mRNA vaccines after intradermal electroporation might be increased by strategies that temper their inherent innate immunogenicity.

## 1. Introduction

Zika virus (ZIKV) is a mosquito-transmitted flavivirus that was isolated for the first time in 1947 in the blood of a sentinel Rhesus monkey in the Zika forest of Uganda. Like other flaviviruses, ZIKV is an enveloped, positive single-stranded RNA virus. Its full-length genome, which encodes three structural proteins (C, prM, and E) and seven non-structural proteins (NS1, 2A, 2B, 3, 4A, 4B, and 5), is translated as a large polyprotein that is cleaved by viral and host proteases [1]. The ZIKV envelope comprises the membrane (M) and envelope (E) proteins. The M protein is derived from the pre-membrane (prM) protein, which is cleaved during virus maturation into the M protein. The prM protein acts as a chaperone and anchors the E protein in the lipid layer [2].

Until recently, ZIKV infections were associated with a mild self-limited illness, characterized by fever, rash, fatigue, headache, conjunctivitis, and myalgia. The first signs that ZIKV was more dangerous than originally thought were seen during the outbreak in French Polynesia (2013/2014), where the ZIKV epidemic was linked with a surge in cases of Guillain–Barré syndrome [3,4,5]. Later on, in 2016, the large ZIKV epidemic in South America pointed out that ZIKV was also responsible for fetal malformations, such as microcephaly, cerebral calcifications, eye defects, and hearing loss [6]. In Rio de Janeiro (Brazil), 42% of infants born from ZIKV-infected women had abnormal clinical or brain imaging findings [7], while in French territories in the Americas, this percentage was much lower [8]. During the outbreak in Brazil, more than 2800 babies with ZIKV-related fetal malformation were born [6,9]. Furthermore, there are indications that exposed but asymptomatic babies born from ZIKV-infected mothers may have neurological abnormalities, such as vision and intellectual impairments, that are not readily detected [10]. 

Although the ZIKV outbreak in South America faded at present, the transmission still progresses at low and steady levels and recurrence is expected in the coming years [11,12]. Furthermore, high mutation rates found in ZIKV can favor the genesis of new strains [13]. The severity of the disorders caused by ZIKV and the real risk of new ZIKV outbreaks underpins the need of an effective and safe vaccine. During the last years, several ZIKV vaccines based on inactivated or attenuated ZIKV, virus-like particles, viral, or non-viral vectors (DNA and mRNA) have been tested in mice and non-human primates [14,15,16,17,18]. Some of these ZIKV vaccines are currently evaluated in clinical trials [19]. The E protein, which is exposed at the surface of the viral particle, is the major target of neutralizing antibodies. Hence, all subunits of ZIKV vaccines are based on this antigen. Moreover, it has been shown that expression of the prM-E polyprotein of flaviviruses, like ZIKV, by (non-)viral vectors results in the secretion of highly immunogenic virus-like particles (VLPs) that expose the E protein at their surface [2,17]. Although vaccines based on viral vectors have a high potency, they have important drawbacks such as a labor-intensive large-scale production process, the possible neutralization of the viral vector by a pre-existing or induced adaptive immune response, and the risk of insertional mutagenesis when integrating vectors are used [20]. Therefore, DNA and mRNA vaccines have been studied as possible alternatives. However, DNA vaccines have shown only limited success in clinical trials [21,22,23,24]. Moreover, there is also the potential risk that they may integrate in the genome [25]. Therefore, during the last decade, mRNA vaccines have emerged as safer and more potent alternatives because, unlike DNA vaccines, they do not need to cross the nuclear membrane to be effective [26,27]. Indeed, mRNA delivery in the cytosol suffices to initiate translation into the desired protein. This cytosolic delivery can be increased by non-viral carriers [28]. However, a drawback of non-viral carriers is that they may cause in vivo toxicity and make the commercial production and registration of mRNA therapeutics more complicated [29,30,31,32]. A humoral immune response after local administration (i.m. or i.d.) of naked mRNA vaccines has only been obtained with self-replicating mRNAs (sr-mRNA), albeit with a high variability and lower efficacy than lipid nanoparticle (LNP)-formulated sr-mRNA [33,34].

In this work, we designed and evaluated, for the first time, a naked sr-mRNA ZIKV vaccine encoding the ZIKV prM-E. Intradermal electroporation of 1 µg of this unformulated ZIKV vaccine in IFNAR1 knockout C57BL/6 mice (IFNAR1^-/-^ mice) elicited highly reproducible antibody titers that were comparable to previously reported titers after vaccination with formulated non-replicating and replicating mRNA ZIKV vaccines [14,35,36,37]. Moreover, all vaccinated IFNAR1^-/-^ mice were protected against ZIKV challenge. However, in wild-type (WT) C57BL6 mice, the vaccine elicited much lower and highly variable antibody titers. Our data indicate that the elicited type I IFNs after intradermal electroporation of the sr-prM-E-mRNA vaccine are responsible for this weak and highly variable humoral immune response in these mice. This is in line with the study of Pepini et al. who also found that the elicited type I IFNs by a formulated sr-mRNA vaccine against respiratory syncytial virus had a negative impact on the antibody titers. They concluded that the type I IFNs suppressed the expression of the antigen and that this resulted in lower antibody titers. However, we found indications that the elicited type I IFNs might also have a direct negative effect on the induced immune cells after sr-mRNA vaccination. 

## 2. Materials and Methods 

### 2.1. Mice

Female BALB/c mice (6–8 weeks old) were purchased from Janvier (France). Heterozygous IFN-β reporter (IFN-β^+/Δβ-luc^) mice [38,39] and IFNAR1 knockout (IFNAR1^-/-^) mice were kindly provided by Prof. Johan Grooten (Department of Biomedical Molecular Biology, Ghent University), and Prof. Roosmarijn E. Vandenbroucke (Department of Biomedical Molecular Biology, Ghent University), respectively. The IFNAR1^-/-^ mice were backcrossed for over 10 generations into a homogeneous C57BL6/J background. All transgenic mice were bred in house and kept in individually ventilated cages with free access to feed and water. Mice experiments were approved by the Ethics Committee of the Faculty of Veterinary Medicine, Ghent University (No. EC2016/86 and No. EC2017/39). Experiments with ZIKV were strictly conducted under Biosafety Level 2 (BSL-2) containment conditions.

### 2.2. Cells and Viruses

Baby hamster kidney cells (BHK-21) and Vero cells were purchased from American Type Culture Collection (ATCC) and cultured at 37 °C and 5% CO_2_ in DMEM or EMEM, respectively, supplemented with 10% inactivated fetal calf serum and 100 IU/ml penicillin/streptomycin. ZIKV strain MR-766 was produced at the Institute of Tropical Medicine Antwerp (Belgium) and the infectious titers, expressed as 50% tissue culture infective dose (TCID_50_) values, were determined in Vero cells using an endpoint dilution assay as described by Reed and Muench [40].

### 2.3. Synthesis of Self-Replicating mRNA Encoding ZIKV PrM-E or Luciferase

The synthesis of the self-replicating mRNAs (sr-mRNAs) was performed by in vitro transcription (IVT) as described [41,42]. Briefly, the sequence of the ZIKV prM-E fusion protein of the Brazilian Rio-S1 ZIKV strain (GenBank No. KU926310.1, base 473 to 2488) or firefly luciferase (LUC) was cloned into the pTK155 plasmid using Gateway Cloning (Invitrogen). The signal peptide of Japanese encephalitis virus (JEV) was placed in front of the ZIKV prM-E sequence. The pTK155 plasmid was a kind gift of dr. Tasuku Kitada and Prof. Ron Weiss (Massachusetts Institute of Technology, USA) and is derived from Venezuelan Equine Encephalitis Virus (VEEV) strain TC-83 containing a point mutation in the 5’UTR (A3G) and in the nsP2 leading to an amino acid substitution (Q739L). The engineered plasmids were sequenced to ensure that they contained the correct inserts. To produce the VEEV-based sr-prM-E-mRNA and sr-LUC-mRNA, the plasmids were transformed into competent *Escherichia coli* (*E. coli*) bacteria and cultured in Luria broth (Invitrogen, Massachusetts, USA). After 16 h, the bacteria were lysed and the plasmids were purified using the Plasmid Plus Midi kit (Qiagen, Germany). Subsequently, the plasmids were linearized with I-SceI endonuclease (NEB, Massachusetts, USA) and sr-mRNA were synthetized by IVT with the MEGAscript T7 Transcription kit (Life Technologies, Massachusetts, USA). After IVT, the sr-mRNAs were purified and post-transcriptionally capped using ScriptCap m7G Capping System and the 2’-O-Methyltransferase kit (Cellscript, Wisconsin, USA) to obtain cap1. After capping, the sr-mRNAs were again purified using the RNeasy® Mini kit (Qiagen, Germany). A Poly(A) tailing was not required as a 40 nucleotide long poly(A) was encoded in the linearized plasmid template. The quantity and quality of the sr-mRNAs were determined using a Nanodrop spectrophotometer (Thermo Fisher Scientific, Massachusetts, USA). The sr-mRNAs were stored at −80 °C. 

### 2.4. In Vitro Analysis of ZIKV E Protein Expression

BHK-21 cells were seeded in a 24-well plate (1 × 10^5^ /well) and transfected at 70% confluency with 500 ng of sr-mRNA encoding ZIKV prM-E or LUC. Transfections were performed with lipofectamine MessengerMax (Invitrogen) following the instructions of the manufacturer. Twenty-four hours after transfection, culture supernatants were collected, and cells were subsequently lysed with RIPA buffer for 1 h on ice. Then, 20 µl cell lysate samples were subsequently supplemented with an equal volume of 2 × SDS-PAGE loading Buffer (4% SDS, 20% glycerol, 200mM DTT, 0.01% bromophenol blue, and 0.1 M Tris HCl, pH 6.8) and incubated for 10 min at 100 ºC before the loading of 15 µl samples on SDS-PAGE gels. Following gel electrophoresis, the proteins were transferred onto PVDF membranes by electroblotting (1 h at 100 V). Next, the membranes were blocked overnight at 4 ºC with 5% non-fat milk in PBS with 0.2% Tween-80 (PBST). Membranes were then incubated with mouse anti-ZIKV-E protein monoclonal antibody (BioFront Technologies, FL, USA) at 1:5000 for 2 h followed by three washes with PBST. After that, the membranes were incubated with a 1:3000 dilution of HRP-conjugated goat anti-mouse lgG (H+L) (GeneCopoeia) for 1 h at room temperature followed by three washes. The membranes were analyzed using ECL western blotting detection kit (Thermo Scientific) and pictured by the ChemiDoc XRS+ system (Bio-rad). For the dot blots, 3 µl of undiluted or 10-fold and 100-fold-diluted supernatant was added directly to PVDF membranes. For the visualization of the ZIKV E protein on these membranes, the same procedure was followed as described for the Western blotting. 

### 2.5. Preparation of Formalin-Inactivated ZIKV Vaccine

The formalin-inactivated ZIKV vaccine (FI-ZIKV) used in this study was prepared using the same conditions as described previously [15]. In more details, a ZIKV isolate from the Dominican Republic (GenBank KU853012, a kind gift from Luisa Barzon, University of Padova, Italy) was grown in Vero cells and harvested after 4 days, followed by low speed centrifugation to remove cell debris and subsequent ultracentrifugation through a 5% glycerol cushion. The pellet was subsequently resuspended in PBS. To inactivate the purified ZIKV, the material was incubated with formalin (0.05%) at 22 °C for 7 days. Subsequently, formalin was removed via dialysis. The inactivation of the virus was confirmed by passaging the material on Vero cells and monitoring the development of a cytopathic effect.

### 2.6. Vaccination Experiments

Female IFNAR1^-/-^ mice, having a C57BL/6 background, or WT C57BL/6 and BALB/c, were vaccinated by two consecutive intradermal electroporations of the sr-prM-E-mRNA ZIKV vaccine or the sr-LUC-mRNA (control). The dose of the sr-prM-E-mRNA ZIKV vaccine, sr-LUC-mRNA control (1 or 10 µg in 50 µl), or FI-ZIKV (1 µg in 50 µl) was split over the left and right flanks of the mice. Electroporation was performed immediately after each injection with a 2-needle array electrode containing 4 needles per row of 4 mm (AgilePulse, BTX Harvard Apparatus, Massachusetts, USA) as previously described [43]. The electroporation involved two short high-voltage pulses of 450 V with duration of 0.05 ms and an interval of 300 ms followed by eight long low-voltage pulses of 100 V with duration of 10 ms and an interval of 300 ms.

### 2.7. Innate Immune Response and Expression

The evoked innate immune response after intradermal electroporation of the sr-prM-E-mRNA vaccine was monitored during 14 days in IFN-β reporter mice (IFN-β^+/Δβ-luc^) using the same doses and administration procedures as in the vaccination experiments. We also compared the expression profile of the sr-LUC-mRNA after intradermal electroporation in WT and IFNAR1^-/-^ C57BL/6 mice. Further, 1 or 10 µg of the sr-LUC-mRNA was intradermally electroporated and the luciferase expression was followed for 28 days. To measure the in vivo luciferase expression, mice were subcutaneously injected with 200 μl D-luciferin (15 mg/ml, Gold Biotechnology, USA) and 12 min later, the in vivo bioluminescence signal was recorded using an IVIS Lumina II (PerkinElmer, USA).

### 2.8. Zika Virus-Specific Antibody Titers

ZIKV E protein-specific antibody titers were determined using the mouse ZIKV ELISA kit (Alpha Diagnostic International, TX, USA). In more details, 96-well plates that were pre-coated with ZIKV E protein were equilibrated for 5 min at room temperature with 300 μl of the provided wash buffer. Subsequently, two-fold serial dilutions of the serum samples were made (starting from a 50-fold dilution) and 100 µl of these dilutions was added per well along with the calibration standards. After 1 h of incubation at room temperature, the plates were washed four times with wash solution. Next, 100 μl of anti-mouse IgG HRP-conjugate working solution was added to the wells and incubated at room temperature. After 30 min, the wells were washed five times and subsequently incubated with 100 μl of 3,3’,5,5’-Tetramethylbenzidine (TMB) substrate at room temperature. The enzymatic conversion of TMB was stopped after 15 min by adding 100 μl of stop solution and the absorbance was measured at 450 nm in a Biochrom EZ 400 microplate reader (Biochrom, England). The antibody endpoint titers were defined as the highest reciprocal dilution with an absorbance that was at least two times the background (obtained with serum of unvaccinated mice).

### 2.9. Zika Virus-Specific Cellular Immune Response

ZIKV-specific CD4^+^ and CD8^+^ T cells responses were determined by intracellular cytokine staining with flow cytometry. In more detail, splenocytes were isolated four weeks after the boost and stimulated in 96-well plates (1×10^6^ cells/well) with 2 μg/ml of overlapping 15-amino-acid peptides covering the ZIKV E protein (JPT, Berlin, Germany) in 1640 RPMI medium. After 1 h of stimulation at 37 °C 0.3 µl of eBioscience™ protein transport inhibitor cocktail (Brefeldin A 5.3 mM + Monensin 1 mM, eBioscience) was added to 150 µl of stimulated splenocytes, and the samples were further incubated for 5 h at 37 °C. Splenocytes were then harvested, washed with cold PBS, treated with mouse BD Fc Block™ (BD Biosciences), and stained with anti-CD3-APC/CD4-PerCP/CD8-Alexa Fluor 488 antibodies (clones 145-2C11, RM4-5 and 53-6.7, Biolegend) for 30 min at 4 °C according to manufacturer’s instructions. Subsequently, the cells were fixed and permeabilized with the Fixation/Permeabilization buffer (eBioscience) for 30 min at 4 °C before intracellular staining with anti-IFN-γ-PE antibody (clone XMG1.2, Biolegend) for 30 min at room temperature. All the samples were finally washed and stored at 4 °C until analysis using a Cytoflex flow cytometer (Beckman Coulter). Single and live cells were gated and 300,000 events were collected for each sample. Samples treated with Cell Stimulation Cocktail (eBioscience) served as positive controls and unstimulated samples as negative controls. 

### 2.10. Zika Virus Challenge

IFNAR^-/-^ mice were intradermally electroporated with 1 µg of the sr-prM-E-mRNA ZIKV vaccine or sr-LUC-mRNA using a prime-boost vaccination regimen with an interval of two weeks. Two weeks after the boost, all the mice were infected in the footpad with 1000 TCID_50_ of ZIKV MR-766 in 25 μl of PBS. One day before the ZIKV challenge, blood samples were taken to determine ZIKV specific neutralization antibody titers. After ZIKV challenge, the body weight and disease symptoms were monitored daily and a scoring system for weight loss and mobility impairment was used to determine survival rates (supplementary Table S1). Blood samples were collected at day 1, 5, and 7 post-challenge to assess viral RNA loads in plasma.

### 2.11. ZIKV Virus Neutralization Tests

The virus neutralization test (VNT) was performed in Vero cells to measure the ZIKV-specific neutralizing antibodies in the vaccinated mice prior to challenge. Serum samples were heat-inactivated at 56 °C for 30 min and subsequently 3-fold serially diluted in assay medium consisting of EMEM (Lonza) supplemented with 2 mM L-glutamine (Lonza), 100 U/ml Penicillin, 100 µg/ml Streptomycin (Lonza), and 2% fetal bovine serum (Gibco). Next, ZIKV (strain MR766, African lineage) suspension was added to each dilution to obtain 36 TCID_50_ (3 TCID_100_) per 100 µl of virus-serum mixtures. The mixtures were subsequently incubated for 1h at 37 °C in 7% CO_2_. After that, 100 µl of each virus-serum mixture was added to an equal volume of Vero cells containing 1.8 × 10^4^ cells and each serum dilution was tested in 8 replicates. The plates were cultured in a humid incubator at 37 °C (7% CO_2_) and the cytopathic effect (CPE) in the cultures was evaluated microscopically after 1 week. Neutralization titers were determined by counting the number of wells showing absence of CPE, and NT_50_ and NT_90_ were calculated as the highest reciprocal dilution of the serum samples that resulted in, respectively, 50% and 90% inhibition of viral infectivity using the Reed–Muench method [40].

### 2.12. Determination of Zika Virus RNA in Blood

One, five, and seven days after the ZIKV challenge, serum samples (40 µl) were collected from the IFNAR1^-/-^ mice and the RNA was extracted using the QIAamp Viral RNA Mini Kit (Qiagen) according to manufacturer’s instructions. The extracted RNA was then reverse-transcribed into cDNA and ZIKV RNA was determined using the RealStar Zika Virus RT-PCR kit 1.0 (Altona diagnostics) following the manufacturer’s protocol.

### 2.13. Statistical Analyses

All statistical analyses were performed using GraphPad Prism software (version 7.0, GraphPad Software Inc., CA, USA) and data are represented as means ± SEM, unless otherwise mentioned. One-way and two-way ANOVAs with Tukey’s multiple-comparison tests were used to analyze statistically significant differences among different animal groups. Area under the curve (AUC) values were calculated using the equation AUC = ∑(t_i+1_ – t_i_)/2 × (C_i_ + C_i+1_), where t_i_ is the starting time point, t_i+1_ is the finishing time point, C_i_ is the starting value, and C_i+1_ is the finishing value for each measurement over time. A *p*-value of <0.05 demonstrated statistically significant differences (*p < 0.05, **p < 0.01, ***p < 0.001, ****p < 0.0001).

## 3. Results

### 3.1. Construction of a Self-Replicating mRNA Expressing the ZIKV Pre-Membrane-Envelope Polyprotein

**Figure 1:**
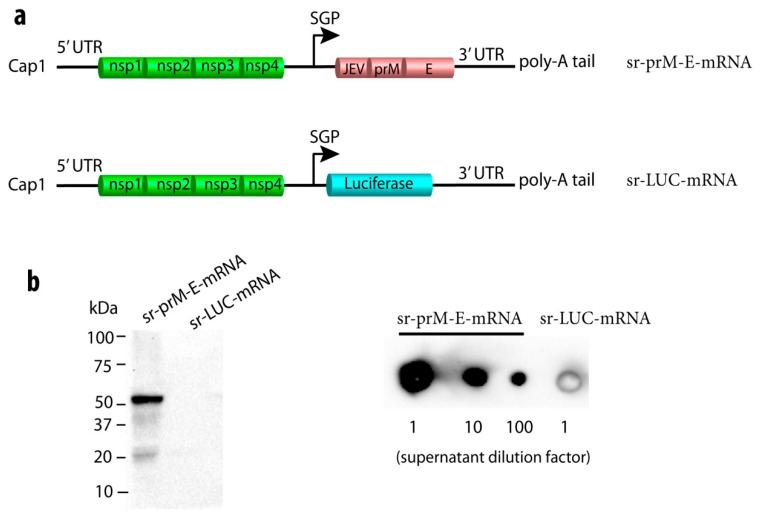
Construction and characterization of a self-replicating (sr) mRNA vaccine against Zika virus (ZIKV). (**a**) Depiction of the sr pre-membrane and envelope (prM-E) mRNA ZIKV vaccine with the Japanese encephalitis virus (JEV) signal sequence in front of the prM-E, and the control sr-mRNA encoding luciferase (sr-LUC-mRNA). The JEV-prM-E and luciferase sequences were codon-optimized. The replication of the sr-mRNA is mediated by the four nonstructural proteins (nsPs) of Venezuelan equine encephalitis virus (VEEV). The UTRs and the subgenomic promotor (SGP) are also derived from VEEV. After transfection of the sr-prM-E-mRNA in baby hamster kidney (BHK)-21 cells, the expression of the ZIKV E protein (~54 kDa) in the cells was detected by Western blot (**b**) and its secretion in supernatant by dot blot (**c**) using monoclonal antibodies against ZIKV E protein and denaturing conditions. BHK cells transfected with sr-LUC-mRNA served as negative controls.

We designed a self-replicating (sr) mRNA encoding the codon-optimized full-length pre-membrane-envelope (prM-E) protein of the Brazilian Rio-S1 ZIKV strain (GenBank No. KU926310.1). The sequence of the Japanese encephalitis virus (JEV) signal peptide was cloned upstream of the prM-E to improve its secretion and cleavage as demonstrated previously [44]. The JEV-prM-E sequence was inserted following the non-structural proteins (nsPs) of Venezuelan equine encephalitis virus (VEEV, strain TC-83) and flanked by the untranslated sequences of VEEV as depicted in Figure 1a. A control sr-mRNA encoding luciferase (sr-LUC-mRNA) was also constructed (Figure 1a). Transfection of BHK-21 cells with the sr-prM-E-mRNA ZIKV vaccine resulted in the production of a correctly processed ZIKV E protein (54 kDa) in the cells and the secretion of the ZIKV E protein in the supernatant, as verified by Western blot and dot blot, respectively (Figure 1b). 

### 3.2. Immunogenicity of the Sr-PrM-E-mRNA ZIKV Vaccine in BALB/c Mice

**Figure 2:**
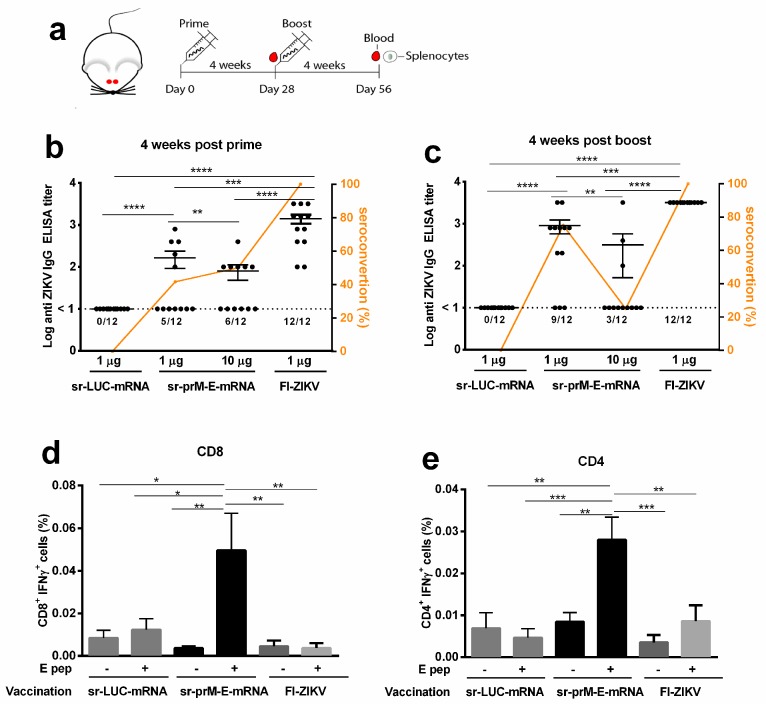
Immunogenicity of the sr-prM-E-mRNA and FI-ZIKV vaccines in BALB/c mice. (**a**) Mice were intradermally electroporated on day 0 and 28 with either 1 or 10 μg of sr-prM-E-mRNA ZIKV vaccine, 1 µg of FI-ZIKV vaccine, or with 1 μg sr-LUC-mRNA. Antibody titers were determined four weeks after the prime (**b**) or boost (**c**) by a ZIKV E protein-specific IgG ELISA (*n* = 12 in each group). The dashed lines indicate the limit of detection of the assay. The lower panels depict ZIKV E protein-specific CD8^+^ (**d**) and CD4^+^ (**e**) T cell responses in mice vaccinated with 1 µg of sr-prM-E mRNA vaccine (*n* = 5 in each group). Mice vaccinated with either 1 µg sr-LUC-mRNA served as negative controls (*n* = 5). T cell responses were determined four weeks after the boost by intracellular staining of IFN-γ in T cells stimulated with a ZIKV E protein peptide pool (E pep +). Mice receiving only alum adjuvant served as controls for the FI-ZIKV vaccinated mice. These mice did not develop ZIKV E protein-specific antibodies or cellular immune responses, as observed for the sr-LUC-mRNA control mice (data not shown). Data are represented as mean ± SEM and were analyzed by ANOVA followed by Tukey’s test.

The immunogenicity of the sr-prM-E-mRNA ZIKV vaccine was first evaluated in BALB/c mice. Animals (12 mice per group) were intradermally electroporated with 1 or 10 μg of the sr-prM-E-mRNA ZIKV vaccine or with 1 μg of the sr-mRNA-LUC control. After four weeks, all mice were boosted using the same dose and procedure (Figure 2a). Mice that received 1 µg of the sr-prM-E-mRNA ZIKV vaccine developed, four weeks after the prime, higher ZIKV E protein-specific IgG antibodies than the mice that received 10 µg of the vaccine (Figure 2b). Seroconversion rates after the prime were around 50% in both groups (Figure 2c). The antibody responses further increased after the boost in the 1 µg group and became significantly higher than the antibody titers in the 10 µg group (Figure 2c). Remarkably, the seroconversion rate dropped in the 10 µg group after the boost from 50% to 25%, while it increased from 42% to 75% in the 1 µg group (Figure 2c). Mice immunized with the sr-LUC-mRNA control did not develop detectable levels of anti-ZIKV E protein antibodies. In addition, the sr-prM-E-mRNA ZIKV vaccine (1 µg group) also elicited ZIKV E protein-specific CD4^+^ and CD8^+^ T cell responses as assessed by intracellular staining of IFN-γ in splenocytes, which were isolated four weeks after the boost (Figure 2d,e). These responses were significantly higher than the background responses observed in mice vaccinated with the control sr-LUC-mRNA. Interestingly, intradermal vaccination of BALB/c mice with formalin-inactivated ZIKV (FI-ZIKV) vaccine in a prime-boost setting elicited high anti-ZIKV E protein-specific titers but failed to induce a ZIKV E protein-specific cellular immune response (Figure 2).

### 3.3. Immunogenicity of the Sr-PrM-E-mRNA ZIKV Vaccine in IFNAR1^-/-^ and WT C57BL/6 Mice

**Figure 3:**
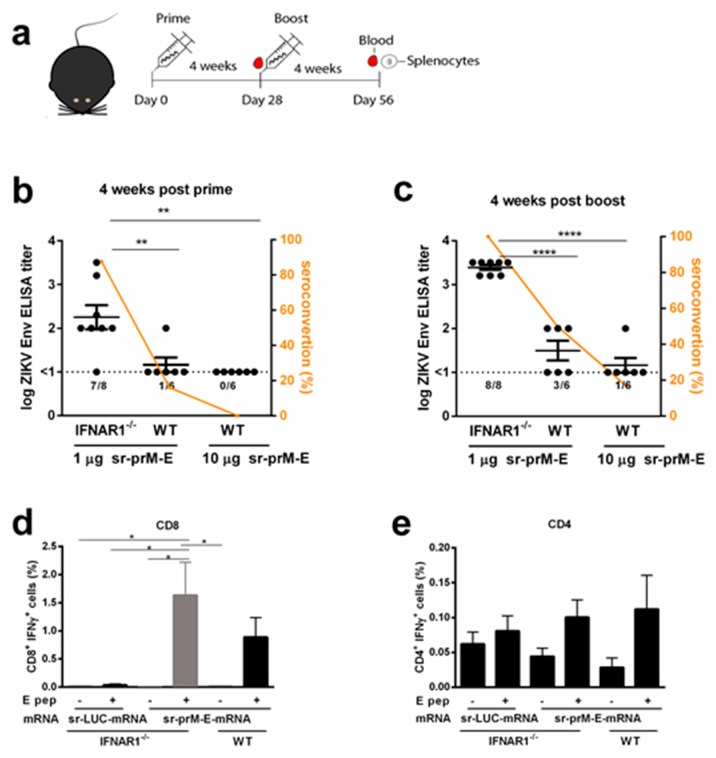
Immunogenicity of the sr-prM-E mRNA vaccine in IFNAR1^-/-^ mice. (**a**) C57BL/6 wild-type (WT) and IFNAR1^-/-^ mice were intradermally electroporated on day 0 and 28 with 1 μg of sr-prM-E-mRNA ZIKV vaccine. An additional group of WT mice was vaccinated in a similar way with a tenfold higher dose of the vaccine. Antibody titers in IFNAR1^-/-^ and WT mice were determined four weeks after the prime (**b**) or boost (**c**) by a ZIKV E-protein specific IgG ELISA (*n* = 6 or 8). The dashed lines indicate the limit of detection of the assay. The lower panels show ZIKV E-protein specific CD8^+^ (**d**) and CD4^+^ (**e**) T cell responses in IFNAR1^-/-^ and WT mice receiving 1 µg sr-prM-E mRNA vaccine or sr-LUC-mRNA. T cell responses were determined four weeks after the boost by intracellular staining of IFN-γ in T cells stimulated with a ZIKV E protein peptide pool (E pep +) (*n* = 6 or 8). All data are represented as mean ± SEM and were analyzed by ANOVA followed by Tukey’s test.

It was reported that BALB/c mice are resistant to infection with African and Asian ZIKV isolates [45]. Also, in our hands, BALB/c mice did not develop disease signs or detectable viremia upon subcutaneous inoculation of up to 10^4^ TCID_50_ of the ZIKV strain MR-766 (data not shown). Therefore, to further evaluate the efficiency of our sr-prM-E-mRNA ZIKV vaccine, the ZIKV susceptible IFNAR1^-/-^ C57BL/6 mice were used. WT and IFNAR1^-/-^ C57BL/6 mice were immunized with 1 µg sr-prM-E-mRNA ZIKV vaccine using the same vaccination protocol as for the BALB/c mice (Figure 3a). Remarkably, after the prime and boost, IFNAR1^-/-^ mice displayed significantly higher anti-ZIKV E protein IgG titers and seroconversion rates than WT C57BL/6 mice (Figure 3b). After the booster vaccination, a tenfold increase in antibody titers and a 100% seroconversion was obtained in IFNAR1^-/-^ mice (Figure 3c). In contrast, in WT C57BL/6 mice, antibody titers and seroconversion rates did not increase much after the boost and remained extremely low in comparison to IFNAR1^-/-^ and BALB/c mice immunized with the same dose (Figure 2c and Figure 3c). Moreover, increasing the dose of the sr-prM-E-mRNA ZIKV vaccine in the WT C57BL/6 mice from 1 to 10 µg resulted in lower antibody titers and seroconversion rates (Figure 3b,c). The ZIKV E-protein specific CD8^+^ T cell responses elicited by the sr-prM-E-mRNA ZIKV vaccine were in IFNAR1^-/-^ mice about three and two-fold higher than in BALB/c and WT C57BL/6 mice, respectively (Figure 3d and Figure 2d). In contrast, the induced ZIKV E-protein specific CD4^+^ T cell responses were very low in IFNAR1^-/-^ and WT C57BL/6 mice and barely above the background responses of control-vaccinated mice (Figure 3e). 

### 3.4. The Interferon Response After Sr-PrM-E-mRNA ZIKV Vaccination Negatively Affects Antibody Titers

**Figure 4:**
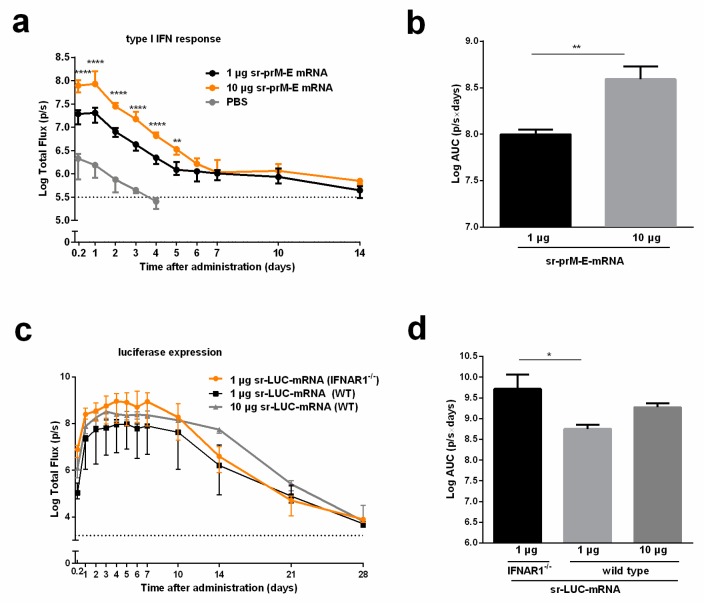
Interferon response after intradermal electroporation of the sr-prM-E mRNA ZIKV vaccine and effect of IFNAR1 on the expression of sr-LUC-mRNA. (**a**) IFN-β luciferase reporter (IFN-β^+/Δβ-luc^) mice were intradermally electroporated with 1 or 10 μg of sr-prM-E-mRNA ZIKV vaccine and the type I interferon response was monitored by measuring the bioluminescent signal at the injection spot for 14 days. The AUC of the curves in (a) are shown in (**b**) (*n* = 3). (**c**) C57BL/6 WT and IFNAR1^-/-^ mice were intradermally electroporated with 1 or 10 μg sr-LUC-mRNA and the luciferase expression was determined by measuring the bioluminescent signal at the injection spot for 28 days. The results of the statistical analysis of the data shown in (**c**) can be found Table S2. The AUC of the curves in (c) are shown in (**d**) (*n* = 4). Data are represented as median with interquartile range and statistical analysis was performed using ANOVA followed by Tukey’s test.

The introduction of a synthetic mRNA into mammalian cells may elicit an innate immune response that results in the release of type I IFNs and inhibition of mRNA translation [27]. Using IFN-β luciferase reporter (IFN-β^+/Δβ-luc^) mice, we confirmed that our sr-prM-E-mRNA ZIKV vaccine evokes a strong and dose-dependent innate immune response directly after intradermal electroporation that gradually decreases after 24 h (Figure 4a,b). Due to the impairment of the late-phase IFN response in IFNAR1^-/-^ mice, a higher translation of sr-mRNAs is expected, as they will elicit a much weaker type I IFN response. We confirmed this hypothesis and found that IFNAR1^-/-^ mice produced 10 times more luciferase proteins than WT C57BL6 mice after intradermal electroporation of sr-LUC-mRNA (Figure 4c,d). However, this difference in sr-mRNA-mediated protein expression might not be the only possible explanation for the difference in anti-ZIKV immune responses between these two mice strains. Indeed, 10 µg of sr-LUC-mRNA in WT C57/BL6 mice caused a similar luciferase expression as 1 µg of sr-LUC-mRNA in IFNAR1^-/-^ mice (Figure 4c,d), but the elicited antibody titers after vaccination with 10 µg sr-prM-E-mRNA ZIKV vaccine in WT C57BL6 mice were much lower than the titers elicited by 1 µg of the vaccine in IFNAR1^-/-^ C57BL6 mice (Figure 3b,c). To evaluate the impact of mRNA replication in the induction of IFN- β signaling, we constructed a non-replicative truncated sr-LUC-mRNA. The mRNA transfer in IFN-β^+/Δβ-luc^ mice confirmed, again, a dose-dependent IFN response and highlighted that an early stage of IFN stimulation was independent of mRNA replication, with peaking at 24 h and fading to background threshold after seven days (Figure S1).

### 3.5. Protective Efficacy of the Sr-PrM-E-mRNA ZIKV Vaccine in IFNAR1^-/-^ Mice

**Figure 5:**
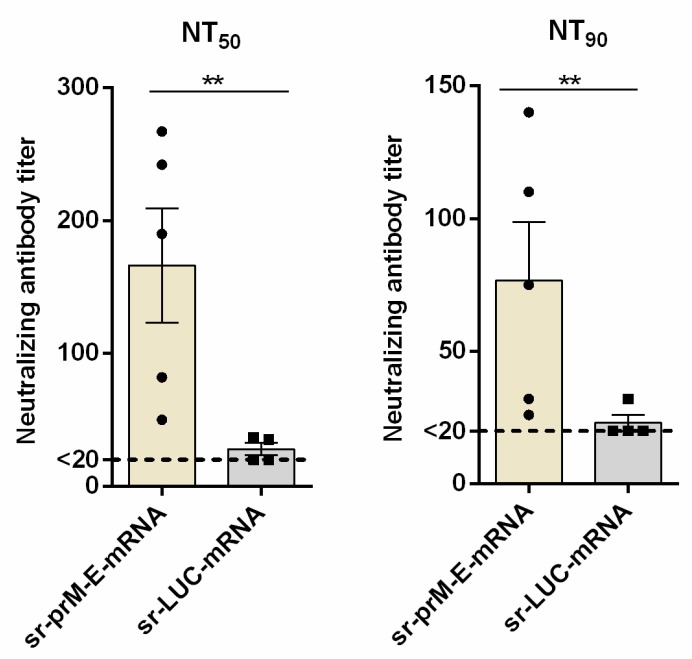
Zika virus neutralizing antibody titers after vaccination of IFNAR1^-/-^ mice with the sr-prM-E mRNA vaccine. Mice were intradermally electroporated on day 0 and 14 with 1 μg of sr-prM-E-mRNA ZIKV vaccine or sr-LUC-mRNA and two weeks after the boost, ZIKV-specific neutralizing antibody titers were determined by a neutralization test (NT). The neutralizing antibody titers are expressed as the reciprocal of the endpoint serum dilution that neutralized the challenge virus by (**a**) 50% (NT_50_) or (**b**) 90% (NT_90_). The lower limit of NT quantification was 20 and is represented by the dashed line (*n* = 5 sr-prM-E-mRNA group and *n* = 4 sr-LUC-mRNA group).

**Figure 6:**
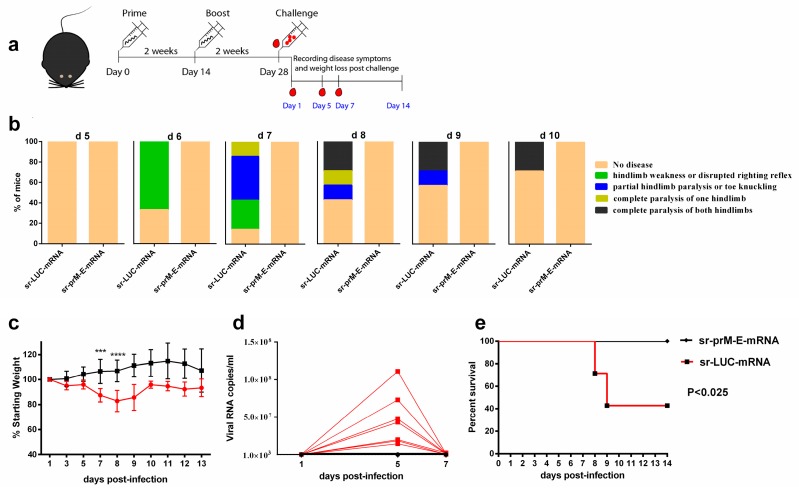
Protective efficacy of the sr-prM-E mRNA vaccine in IFNAR1^-/-^ mice. (**a**) Mice were intradermally electroporated on day 0 and 14 with 1 μg of sr-prM-E-mRNA ZIKV vaccine or sr-LUC-mRNA. Two weeks after the boost they were challenged by injecting 1000 TCID_50_ of ZIKV (MR-766) in the footpad (*n* = 7); (**b**) The disease symptoms were daily recorded from day 5 to day 10 after ZIKV challenge and (**c**) the percentage of weight loss relative to the body mass just before ZIKV infection was calculated; (**d**) the ZIKV RNA loads in plasma were determined by qRT-PCR. The dashed line indicates the limit of detection of the assay; (**e**) survival rates were determined based on the scoring system described in Table S1.

Finally, we determined whether the sr-prM-E-mRNA ZIKV vaccine was able to confer protection against ZIKV infection. To that end, IFNAR1^-/-^ mice (seven mice per group) were immunized with 1 μg of sr-prM-E-mRNA ZIKV vaccine or sr-LUC-mRNA control using a prime-boost regimen with a two-week interval, as the older IFNAR1^-/-^ mice (over three months old) were less susceptible to ZIKV infection [46]. Using a virus neutralization test, we confirmed the presence of ZIKV-specific neutralizing antibodies in the vaccinated mice two weeks after the boost and just before challenge (Figure 5a,b). Subsequently, all mice were challenged with 1000 TCID_50_ of ZIKV MR-766 via the footpad and disease symptoms, weight loss, viremia, morbidity, and mortality were carefully monitored (Figure 6). Control mice started to lose weight on the third day following ZIKV challenge (Figure 6c). On the sixth day post-infection, the control mice also started to show disease symptoms (Figure 6b) that further exacerbated until day 8 post ZIKV challenge. After that point, control mice that survived the ZIKV infection started to recover. However, their weight stayed below that of vaccinated mice during the whole experiment. The observed disease symptoms in control mice were in accordance with the high viral loads in their plasma (Figure 6d). Viremia peaked at day 5 and returned to baseline at day 7 post-infection (Figure 6d). In contrast, none of the mice that were immunized with the sr-prM-E-mRNA ZIKV vaccine developed detectable viremia (<1000 copies/ml), disease symptoms, or weight loss after ZIKV challenge (Figure 6b–d). Upon ZIKV challenge, none of the vaccinated mice died, while four out of seven mice succumbed in the control group (Figure 6e)

## 4. Discussion

In this work, we designed a self-replicating mRNA encoding the prM-E protein of ZIKV. After intracellular delivery of this sr-prM-E-mRNA vaccine, a correctly processed ZIKV E protein was produced and secreted. The immunogenicity of this mRNA-based ZIKV vaccine was subsequently evaluated in three different mouse models using intradermal electroporation. For the in vivo delivery of mRNA vaccines, most preclinical and clinical studies make use of a non-viral carrier e.g., lipid nanoparticles [26,27,47]. Nevertheless, the combination of our naked sr-prM-E-mRNA ZIKV vaccine with local electroporation may hold some important advantages over carrier-mediated delivery, such as a relatively simple commercial production process, a straightforward registration procedure, and less concern about possible toxic effects of the carrier [29,30,31,32]. Moreover, in vivo electroporation systems that are approved for clinical use in humans, like the Cliniporator® (IGEA, Carpi, Italy), are currently used in many clinics to treat cancer patients with electrochemotherapy [48,49].

In ZIKV-susceptible IFNAR1^-/-^ mice, which have a defective IFN type I signaling, intradermal electroporation of only 1 µg sr-prME-mRNA vaccine elicited highly reproducible antibody titers both in BALB/c mice and IFNAR^-/-^ mice. The induced antibody titers in IFNAR^-/-^ mice were comparable to the titers previously obtained with formulated non-replicating or replicating mRNA ZIKV vaccines [14,36,37]. Furthermore, the sr-prM-E-mRNA vaccine resulted in a 100% seroconversion and provided complete protection against a ZIKV challenge. In BALB/c mice, the elicited mean antibody titers were slightly lower and much more variable than the ones obtained in IFNAR1^-/-^ mice. In addition, 3 out of 12 BALB/c mice did not develop detectable antibodies titers after sr-prM-E-mRNA vaccination. In C57BL/6 mice, the results were more disappointing, with a seroconversion of only 50% and mean antibody titers below 100. 

Using IFN-β reporter mice, we showed that intradermal electroporation of the sr-prM-E-mRNA instantaneously elicits a strong and dose-dependent IFN-β response that peaks after 24 h and subsequently gradually disappears. This rapid response indicates that not the cytosolic replication of the sr-mRNA, but rather the intracellular influx of the sr-prM-E-mRNA is mainly responsible for the elicited type I IFN response. To confirm this hypothesis, we made a replication-deficient sr-LUC-mRNA. Intradermal electroporation of this replication-deficient sr-LUC-mRNA elicited a similar type I IFN response in IFN-β reporter mice as a replication-competent sr-prM-E-mRNA (Supplementary Figure S1). 

Recently, Pepini et al. also reported that an LNP-formulated sr-mRNA vaccine elicited a significantly higher expression and humoral response in IFNAR1^-/-^ mice than in WT mice. These authors reported that the lower expression in WT mice is a result of the type I IFNs elicited by the sr-mRNA vaccine and they assumed that the lower antibody titers in WT mice are due to this lower expression [50]. However, by comparing the expression and the antibody titers of our sr-prM-E-mRNA ZIKV vaccine at different doses in WT and IFNAR1^-/-^ mice we found clear indications that the elicited type I IFNs also negatively impact the humoral response by other mechanisms than by reducing the antigen expression. Intradermal electroporation of our sr-LUC-mRNA at 1 µg in IFNAR1^-/-^ mice or at 10 µg in WT mice resulted in a similar luciferase expression. However, intradermal electroporation of 1 µg sr-prM-E-mRNA in IFNAR1^-/-^ elicited a strong antibody response, while intradermal electroporation of 10 µg sr-prM-E-mRNA in WT mice induced a very weak and variable antibody response. These data indicate that the difference in antigen expression of the sr-prM-E-mRNA in IFNAR1^-/-^ and WT mice is not the only reason for the reduced antibody titers. We hypothesize that the elicited type I IFNs not only lowered the expression of our naked sr-mRNA ZIKV vaccine, but also negatively modulated the induced humoral immune response as previously reported in three back-to-back publications in Science Immunology. In these papers, the poor induction of neutralizing antibodies after a lymphocytic choriomeningitis virus (LCMV) infection was attributed to type I IFNs that are induced after cellular entry of LCMV [51]. These papers unraveled several mechanisms by which type I IFNs can interfere with the humoral immune response. Some of these mechanisms are possibly also involved in the type I IFN-mediated blockage of the antibody response after intradermal vaccination with our naked sr-prM-E-mRNA vaccine. Moseman et al. found that the elicited type I IFNs promote a CD8^+^ mediated killing of activated B cells that expose LCMV epitopes [52]. Sammicheli et al. linked type I IFNs with lymph node recruitment of inflammatory monocytes, which prevent, through their NO production, the development of long-lived antibody-secreting cells (ASCs) [53]. Finally, Fallet et al. concluded that the strong type I IFN response after LCMV infections results in short-lived ASCs and that cytokines like IL-10 and TNF-β play a role in this phenomenon [54].

Messenger RNA-based ZIKV vaccines have shown excellent immunogenicity and protection in four previous studies using BALB/c and C57BL/6 mice [14,35,36,37]. One can wonder why type I IFNs did not negatively affect the immunogenicity of these mRNA ZIKV vaccines. Pardi et al. [14] and Richner et al. [35] used LNP formulated modified non-replicating mRNAs, which are known to have a very low inherent innate immune stimulating capacity [27]. A sr-prM-E-mRNA vaccine similar to ours was recently evaluated by Erasmus et al. [36] and Chahal et al. [37]. In contrast to our work, the sr-prM-E-mRNA vaccines in these studies were formulated into nanoparticles and injected intramuscularly instead of intradermally. Indeed, we found that intradermal administration of sr-mRNA induced a significantly higher type I IFN response than intramuscular route at the early stage (Supplementary Figure S2). Additionally, in our sr-prM-E-mRNA vaccine, we used the JEV signal peptide, while Erasmus et al. used a homotypic ZIKV signal peptide that resulted in significantly (up to 100-fold) higher neutralizing antibody titers. These differences may be responsible for the discrepancy with our study. 

Our hypothesis that the elicited type I IFNs suppressed the induction of anti-ZIKV antibodies is further supported by the observation that increasing the dose of our sr-prM-E-mRNA vaccine from 1 to 10 µg resulted in a stronger IFN-β response (Figure 4a) and lower antibody titers (Figure 2b). Additionally, the lower antibody titers and seroconversion rates in C57BL/6 mice (Figure 2b,c versus Figure 3c) are also in agreement with the observation that C57BL/6 mice secrete significantly higher levels of type I IFNs than BALB/c mice [55]. These data indicate that the strength of the elicited type I IFN response is inversely proportional with the induced antibody titers, and that a certain threshold of type I IFNs might be needed before they can suppress the humoral immune response. Therefore, strategies that decrease the inherent innate immunity of sr-mRNA are expected to enhance the efficacy of naked sr-mRNA vaccines after intradermal electroporation. 

The formalin-inactivated ZIKV (FI-ZIKV) vaccine that we tested in our work evoked slightly higher ZIKV specific antibody titers but failed to induce a cellular immune response. In contrast, the sr-prM-E-mRNA ZIKV vaccine induced a robust CD8^+^ T cell response that largely exceeded previously reported CD8^+^ responses with other ZIKV vaccines [14,15]. The CD8^+^ T cell response was higher in IFNAR1^-/-^ mice than in WT mice. This is in agreement with the work of De Beuckelaer et al. who also found a better cellular immune response in IFNAR1^-/-^ mice after immunization with a formulated mRNA vaccine [39]. Although the humoral response is considered as the most important in the protection against ZIKV infection, it has been shown that cellular immunity plays a non-negligible role in controlling ZIKV infection [56,57]. Adoptive transfer of CD8^+^ T cells was shown to reduce viral burden and mortality after ZIKV infection. Additionally, CD8^+^ T cells also provide protection against ZIKV infection during pregnancy and can prevent antigen-induced antibody-dependent enhancement of dengue disease in mice vaccinated against ZIKV [57,58].

The most devastating effects of a ZIKV infection are microcephaly and other distortions of the growing fetuses. Consequently, it is meaningful to determine whether our ZIKV vaccine can protect against these congenital malformations in pregnant mice. In a preliminary study, we observed that unvaccinated mice that were challenged with ZIKV shortly after conception had reduced offspring numbers (four pups from three mated females) compared to sr-prM-E-mRNA vaccinated females (17 pups from 4 mated females). Furthermore, the offspring of unvaccinated mice were born death and one of the four pups showed face malformation. These preliminary data might indicate that our vaccine protects fetuses of pregnant mice against ZIKV. However, additional experiments are needed to confirm and underpin these observations.

## 5. Conclusions

In summary, intradermal electroporation of a naked self-replicating mRNA encoding the prM-E of ZIKV elicited humoral and cellular responses in BALB/c and especially in IFNAR1^-/-^ C57BL/6 mice, resulting in complete protection of the latter mice against subsequent ZIKV challenge. In WT BALB/c and C57BL/6 mice, the seroconversion rates and antibody titers were inversely related to the dose of mRNA as well as the induced IFN-β response determined in a reporter mice model. The increased seroconversion and antibody titers in IFNAR1^-/-^ C57BL/6 indicate the negative impact of early IFN response on sr-prM-E-mRNA vaccination. Nevertheless, a robust CD8^+^ T cell immune response was observed in these mice. By comparing the expression and the antibody titers of our self-replicating mRNA ZIKV vaccine at different doses in WT and IFNAR1^-/-^ C57BL/6 mice, we conclude that the elicited innate immune response after intradermal electroporation of our sr-mRNA ZIKV vaccine can impede the humoral immune response by inhibiting the sr-mRNA translation, and possibly also via a negative modulation of the induced humoral immune response.

## Supplementary Materials

The following are available online at https://www.mdpi.com/2076-393X/7/3/96/s1, Figure S1: The effect of replication of mRNA on the induction of type I interferon in IFN-β luciferase reporter mice, Figure S2: The effect of different routes for mRNA delivery on the induction of type I interferon in IFN-β luciferase reporter mice, Table S1: A scoring system for weight loss and mobility impairment was used to determine survival rates following ZIKV challenge, Table S2: The significant differences of Figure 4c.
