# Supplementary material for: Immunogenicity and Protection Efficacy of a Naked Self-Replicating mRNA-Based Zika Virus Vaccine
Source: Vaccines (Basel). 2019 Aug 23;7(3):96. doi: 10.3390/vaccines7030096 (PMC6789535; doi:10.3390/vaccines7030096)
Supplement: Supplementary file 1 [file vaccines-07-00096-s001.pdf]

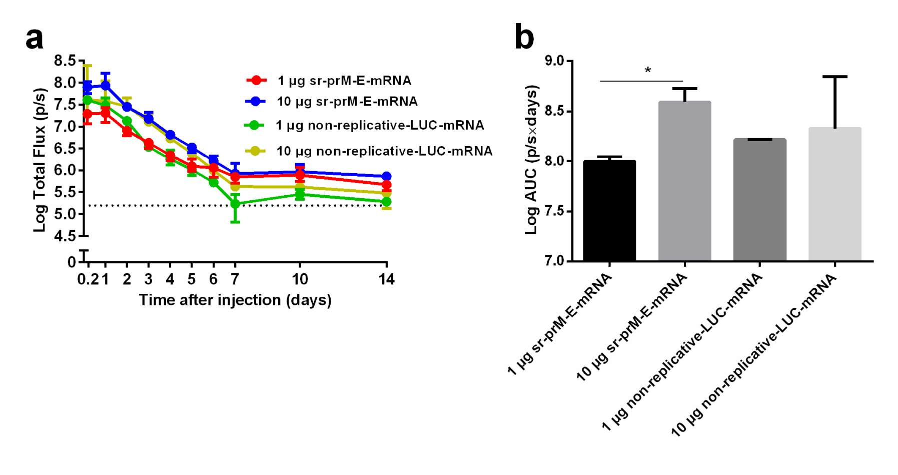

Figure S1. The effect of replication of mRNA on the induction of type I interferon in IFN-β luciferase reporter mice. (**a**) IFN-$\beta^{+/\Delta\beta\text{-luc}}$ mice were intradermally electroporated with 1 or 10 μg of replication-deficient replicon (non-replicative-LUC-mRNA) or replication-competent sr-prM-E-mRNA. The non-replicative-LUC-mRNA was constructed by removal of a fragment of 1404 bp in the sequence encoding nsP2 by the restriction enzyme KpnI and was no longer able to produce luciferase. The type I interferon responses were monitored by measuring the bioluminescent signal at the injection spot for 14 days. The AUC of the curves in (a) are shown in (**b**) (n = 3) (Data are represented as median with interquartile range and were analyzed by ANOVA followed by Tukey's test.).

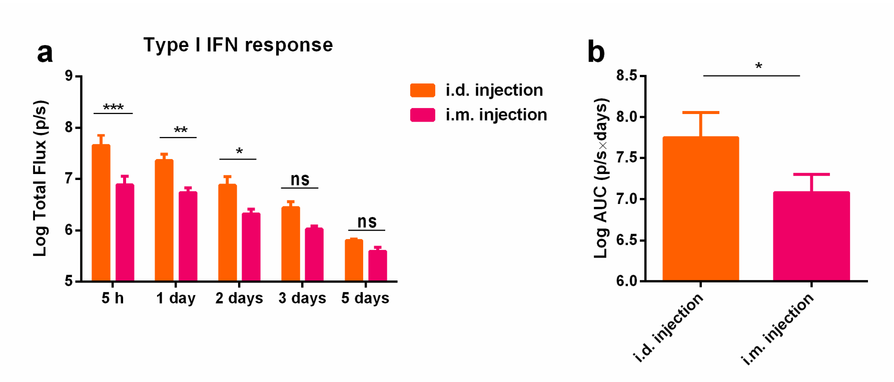

Figure S2. The effect of different routes for mRNA delivery on the induction of type I interferon in IFN-β luciferase reporter mice. (**a**) IFN-$\beta^{+/\Delta\beta\text{-luc}}$ mice were intradermal or intramuscular injected with 1 μg of sr-prM-E-mRNA. The type I interferon responses were monitored by measuring the bioluminescent signal at the injection for 5 days. The AUC of the curves in (a) are shown in (**b**) (n = 4).

Table S1. A scoring system for weight loss and mobility impairment was used to determine survival rates following ZIKV challenge.

Score	Weight Loss	Score	Mobility
1	10%–15% loss of body weight	1	Hindlimb weakness or disrupted reghting reflex
2	15%–20% loss of body weight	2	Partial hindlimb paralysis or toe knuckling
3	20%–25% loss of body weight	3	Complete paralysis of one hindlimb
4	>25% loss of body weight	4	Complete paralysis of both hindlimbs

Mice that lost > 25% of their initial body weight, or showed complete paralysis of both hindlimbs, or that had a combined total score of 4 or more were euthanized and considered dead.

Table S2. The significant differences of Figure 4c.

**Days After Administration**	**0.2**		**1**		**2**		**3**		**4**		**5**	
**Significance (*P*-value)**	**1 μg (WT)**	**10 μg (WT)**	**1 μg (WT)**	**10 μg (WT)**	**1 μg (WT)**	**10 μg (WT)**	**1 μg (WT)**	**10 μg (WT)**	**1 μg (WT)**	**10 μg (WT)**	**1 μg (WT)**	**10 μg (WT)**
**1 μg (KO)**	**** (<0.0001)	** (0.0046)	**** (<0.0001)	* (0.0486)	**** (<0.0001)	ns	**** (<0.0001)	ns	**** (<0.0001)	ns	**** (<0.0001)	ns
**1 μg (WT)**		**** (<0.0001)		**** (<0.0001)		**** (<0.0001)		**** (<0.0001)		** (0.0012)		** (0.0019)
**Days after administration**	**6**		**7**		**10**		**14**		**21**		**28**	
**Significance (*P*-value)**	**1 μg (WT)**	**10 μg (WT)**	**1 μg (WT)**	**10 μg (WT)**	**1 μg (WT)**	**10 μg (WT)**	**1 μg (WT)**	**10 μg (WT)**	**1 μg (WT)**	**10 μg (WT)**	**1 μg (WT)**	**10 μg (WT)**
**1 μg (KO)**	**** (<0.0001)	ns	**** (<0.0001)	ns	**** (<0.0001)	ns	ns	**** (<0.0001)	ns	*** (0.0002)	ns	ns
**1 μg (WT)**		**** (<0.0001)		*** (0.0001)		**** (<0.0001)		**** (<0.0001)		ns		ns
